# Overexpression of LAPTM4B-35 is a negative prognostic factor in head and neck squamous cell carcinoma

**DOI:** 10.1038/s41598-019-55319-z

**Published:** 2019-12-11

**Authors:** Ulana Kotowski, Lorenz Kadletz, Sven Schneider, Felicitas Oberndorfer, Julia Schnoell, Elisabeth Gurnhofer, Lukas Kenner, Trevor Lucas, Gregor Heiduschka

**Affiliations:** 1 Department of Otorhinolaryngology, Head and Neck Surgery, Medical University of Vienna, Waehringer Guertel 18-20, 1090 Vienna, Austria; 2 Department of Pathology, Medical University of Vienna, Waehringer Guertel 18-20, 1090 Vienna, Austria; 3 Center for Anatomy and Cell Biology, Department for Cell and Developmental Biology, Medical University of Vienna, Schwarzspanierstraße 17, 1090 Vienna, Austria

**Keywords:** Cell biology, Cancer, Translational research, Cancer

## Abstract

Overexpression of LAPTM4B-35 (lysosomal-associated transmembrane protein 4β-35) is associated with a poor prognosis in numerous malignant tumours. Expression patterns and effects of LAPTM4B-35 on head and neck squamous cell carcinomas (HNSCC) are unknown. The aim of this study was to investigate the prognostic relevance of LAPTM4B-35 in HNSCC. Tissue microarrays were constructed with primary tumours and associated lymph node metastases isolated from 127 patients. The expression of LAPTM4B-35 was investigated by immunohistochemistry and the results were correlated with survival data. LAPTM4B-35 in the primary tumour was highly expressed in 47.2% of the patients (60/127). LAPTM4B-35 expression was significantly associated with tumour stage. Moreover, overexpression of LAPTM4B-35 correlated with a significantly worse disease-free survival (10.23 years vs. not reached) and a higher recurrence rate (40.7% vs. 25%). High expression of LAPTM4B-35 in lymph node metastasis was found in 29.2% of cases. In 19.4% of cases, high LAPTM4B-35 expression was observed in both the primary tumour and corresponding lymph node metastases. In conclusion, our data indicates that overexpression of LAPTM4B-35 is associated with poor prognosis and may therefore serve as a new prognostic marker in HNSCC.

## Introduction

More than 500,000 new cases of head and neck cancer are diagnosed worldwide every year^1^. For early stage head and neck squamous cell carcinoma (HNSCC) treatment options include surgery or radiotherapy. However, due to an unclear symptomatic during disease progression, diagnosis is often made at an advanced stage and only multimodal therapy associated with significant side effects is appropriate^2,3^. Despite advances in treatment modalities, prognosis has not significantly improved in recent decades and the 5-year survival rate is currently 65%^4,5^. For treatment of recurrent or metastatic HNSCC, T-cell checkpoint inhibitors targeting programmed cell death protein-1 have emerged. However, only a minority of patients benefit from this therapeutic approach^6,7^. New molecular biomarkers are therefore required to identify high-risk patients in need of high intensity therapeutic approaches and to stratify patients that may profit from specific therapies in novel personalized medicine approaches to oncology.

The lysosomal-associated transmembrane protein 4B (*LAPTM4B*) gene was originally discovered as a transcript expressed in fetal and adult liver cells that is overexpressed in cases of hepatocellular carcinoma^8^. The LAPTM4B-35 protein is overexpressed in a variety of human cancers^9^. Studies in gallbladder carcinoma, pancreatic carcinoma, gastric cancer, breast cancer, endometrial carcinoma, ovarian carcinoma, cervical carcinoma, colorectal carcinoma, prostate cancer and small cell lung cancer have shown that overexpression of LAPTM4B-35 is associated with poor prognosis^10–19^. Further studies had shown that LAPTM4B-35 leads to increased cell proliferation^20^, migration and invasion^21^, inhibition of apoptosis^9^ and promotion of drug resistance^22^. Oncogenic mechanisms mediated by LAPTM4B-35 include activation of the PI3K/AKT signalling pathway^9^. LAPTM4B-35 also inhibits epidermal growth factor receptor (EGFR) intraluminal sorting and lysosomal degradation induced by EGF, leading to enhanced and prolonged EGFR signalling^23^. Other studies have shown that, LAPTM4B-35 mediates EGFR overexpression^24^. Furthermore, LAPTM4B-35 promotes LAT1-4F2hc Leu transporter uptake into lysosomes and activates mammalian target of rapamycin 1^25^. In human regulatory T cells, LAPTM4B-35 is a negative regulator of transforming growth factor-β1 production and may therefore play a role in immune responses^26^.

Although LAPTM4B-35 seems to be a promising marker for a variety of carcinomas, there is currently no clinical data available on protein expression in HNSCC. A very recent study found up-regulation of *LAPTM4B* at the RNA level in HNSCC^27^. The aim of this study was to evaluate LAPTM4B-35 protein as a new prognostic marker for HNSCC in primary tumours and lymph node metastases and correlate results with clinical data.

## Results

### Clinical data

Specimens from 127 patients with HNSCC were analysed (Table 1). If available, lymph node specimens were also evaluated (n = 72). In total, 36 (28.3%) patients had a primary tumour located in the oral cavity, 56 (44.1%) in the oropharynx, 21 (16.5%) in the hypopharynx and 14 (11.0%) in the larynx. Information on human papillomavirus (HPV) status was assessable in 55 (98.2%) patients with oropharyngeal carcinoma and 24 (43.6%) patients were positive. The cohort comprised 98 (77.2%) men and 29 (22.8%) woman. The mean age at the time of diagnose was 57.6 years (median 59 years, range 27–80 years). The mean follow-up was 121.3 months (median 115.7 months). All patients received initial surgical treatment, followed by radiotherapy with a mean dose of 58.4 Gy (median 60 Gy, range 40–70 Gy). Additional chemotherapy was administered postoperatively to 19 (14.9%) patients. According to the classification system of the Union for International Cancer Control (UICC) (7^th^ edition), 3 (2.4%) patients were classified as stage I, 17 (13.4%) as stage II, 30 (23.6%) patients as stage III and 77 (60.6%) as stage IVa. The calculated median overall survival (OS) for all patients was 101.3 months and the median disease-free survival (DFS) survival was 81.8 months.

**Table 1:** Relationship between LAPTM4B-35 expression and clinicopathological features of patients with head and neck cancer.

Variables	Patient number	LAPTM4B-35 expression		*p*-value
		low	high	
Age				0.265
<60	68	39 (57.4%)	29 (42.6%)	
>60	59	28 (47.5%)	31 (52.5%)	
Gender				0.047
Male	98	47 (48.0%)	51 (52.0%)	
Female	29	20 (69.0%)	9 (31.0%)	
Anatomical site				0.096
Oral cavity	36	13 (36.1%)	23 (63.9%)	
Oropharynx	56	35 (62.5%)	21 (37.5%)	
Hypopharynx	21	12 (57.1%)	9 (42.9%)	
Larynx	14	7 (50.0%)	7 (50.0%)	
Lymph node metastasis				0.070
Yes	99	48 (48.5%)	51 (51.5%)	
No	28	19 (67.9%)	9 (32.1%)	
UICC stage				0.017
I	3	0 (0.0%)	3 (100%)	
II	17	13 (76.5%)	4 (23.5%)	
III	30	19 (63.3%)	11 (36.7%)	
IV	77	35 (45.5%)	42 (54.5%)	
N classification				<0.001
0	28	19 (67.9%)	9 (32.1%)	
1	30	16 (53.3%)	14 (46.7%)	
2a	8	2 (25.0%)	6 (75.0%)	
2b	45	19 (42.2%)	26 (57.8%)	
2c	13	10 (76.9%)	3 (23.1%)	
3	3	1 (33.3%)	2 (66.7%)	
T classification				<0.001
1	25	12 (48%)	13 (52%)	
2	68	40 (58.8%)	28 (41.2%)	
3	21	11 (52.4%)	10 (47.6%)	
4a	13	4 (30.8%)	9 (69.2%)	

### Expression of LAPTM4B-35 in HNSCC

**Figure 1:**
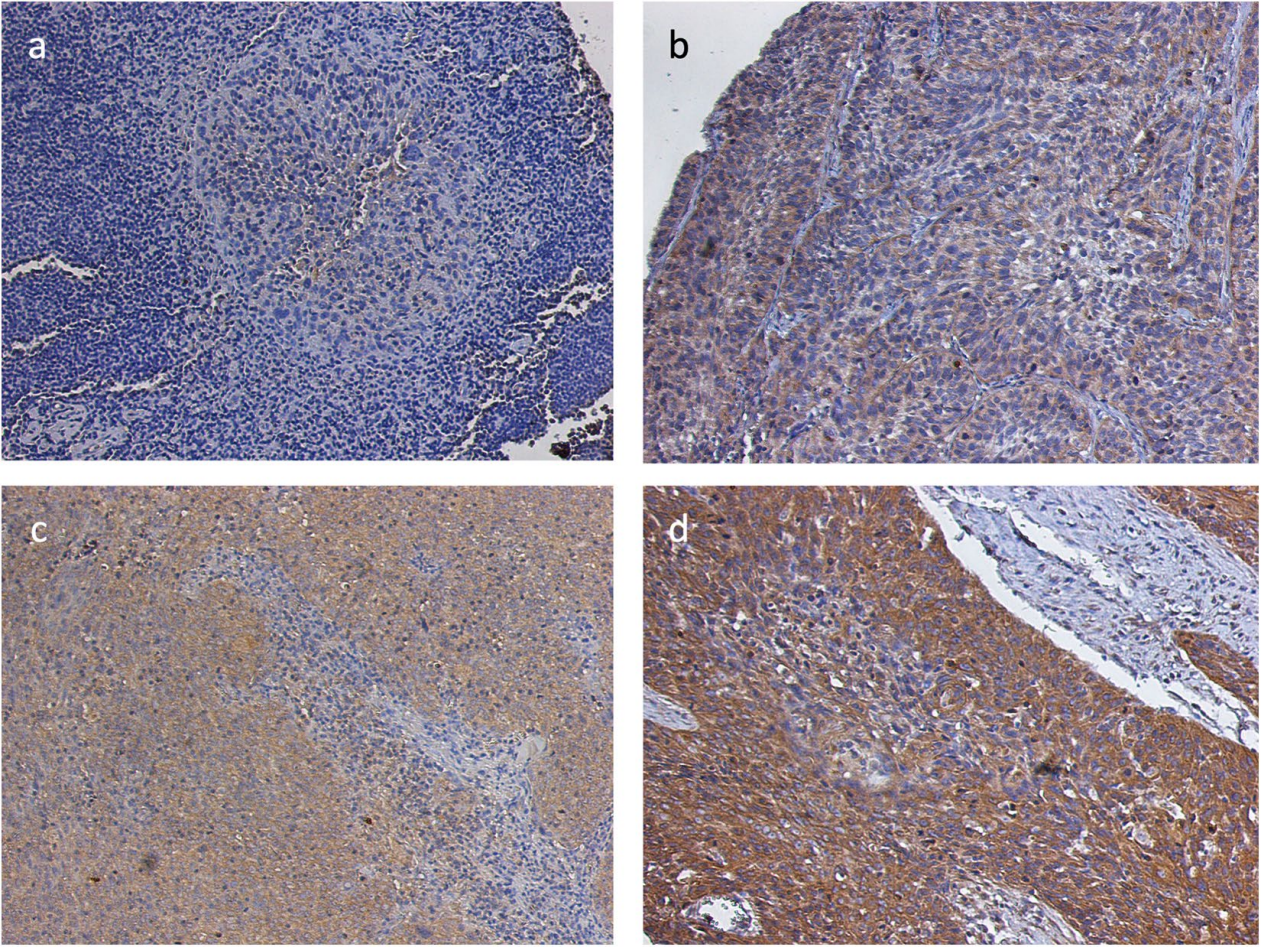
Immunohistochemical expression analysis of LAPTM4B-35 in samples from patients with head and neck squamous cell carcinoma. Examples of tumours with (**a**) no, (**b**) weak, (**c**) moderate and (**d**) strong LAPTM4B-35 expression.

LAPTM4B-35 expression was detected by immunohistochemistry in 120 (94.5%) of 127 primary HNSCC samples. Within the positive samples, 61 (48%) showed weak, 51 (40.2%) moderate and 8 (6.3%) strong visual expression. 7 (5.5%) examined samples showed no staining. Staining for LAPTM4B-35 was always localized in the cytoplasmic compartment of cancer cells (Fig. 1). Adjacent non-cancerous tissue showed no staining. In further statistical analysis, patients were classified into “LAPTM4B-35 low” and “LAPTM4B-35 high”. Samples with no or weak expression were termed “LAPTM4B-35 low” (68/127; 53.6%) and moderately and strongly expressing biopsies were categorized as “LAPTM4B-35 high” (59/127; 46.4%).

### Expression of LAPTM4B-35 stratified by anatomical site and HPV status

We then analysed the expression of LAPTM4B-35 with respect to the anatomical site of HNSCC occurrence. In 63.9% (n = 23/36) of samples from the oral cavity and in 37.5% (n = 21/56) of the oropharyngeal samples, high expression of LAPTM4B-35 was found. High LAPTM4B-35 expression was seen in 42.9% (n = 9/21) of hypopharyngeal carcinomas and 50% (n = 7/14) of laryngeal carcinoma samples (Table 1). In addition, we evaluated whether HPV infection status was associated with differences in LAPTM4B-35 expression. LAPTM4B-35 was highly expressed in 33.3% of HPV positive oropharyngeal tumours and 41.9% of HPV negative tumours. Statistical analysis of the distribution of categorical variables revealed that neither the anatomical subsite (p = 0.096) nor HPV status (p = 0.515) had a significant association with LAPTM4B-35 expression.

### Expression of LAPTM4B-35 in lymph node metastases

Since head and neck tumours frequently metastasize, we also examined the expression of LAPTM4B-35 in lymph node metastases (n = 72). LAPTM4B-35 was classified as high in 21 (29.2%) and low in 51 (70.8%) cases. We then examined whether there were differences in LAPTM4B-35 expression patterns between primary tumours and lymph node metastases. A total of 14 patients (19.4%) had high LAPTM4B-35 expression in both the primary tumour and lymph node metastasis. 23 patients (31.9%) had a high expression only in the primary tumour. In the patient group with low primary tumour LAPTM4B-35 expression, 28 (38.8%) had low expression in the lymph node metastasis and 7 (9.7%) patients showed a high expression profile.

### Correlation between LAPTM4B-35 expression and clinicopathological characteristics

LAPTM4B-35 expression subgroups were then compared to the clinicopathological features of the patient cohort (Table 1). A chi-square test revealed that LAPTM4B-35 expression is significantly linked to UICC stage (p = 0.017). Regarding the T and N classifications, the statistical association was even more significant (p < 0.001).

Furthermore, patients with high LAPTM4B-35 expression showed an increased rate of recurrence. After total follow-up, 40.7% of the patients with high LAPTM4B-35 expression had a recurrence compared to 25% in the LAPTM4B-35 low group. No significant differences in OS between high and low LAPTM4B-35 groups was observed, although more patients in the LAPTM4B-35 high group had died (52.5% compared to 41.2%).

**Figure 2:**
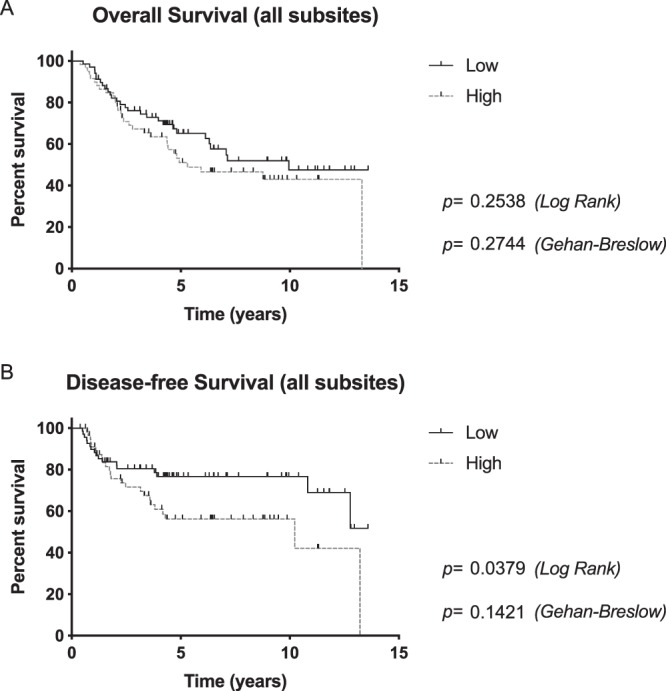
Kaplan-Meier estimates for (**a**) overall survival and (**b**) disease-free survival according to LAPTM4B-35 expression for all head and neck carcinomas. Patients with high LAPTM4B-35 expression had a significantly worse DFS.

Kaplan-Meier estimates of OS and DFS were then evaluated to determine the impact of HNSCC LAPTM4B-35 expression. Calculation of OS showed no statistically significant difference between patients with low and high LAPTM4B-35 expression in the primary tumour (p = 0.2538 [Log Rank], p = 0.2744 [Gehan-Breslow]). Nevertheless, the median OS of patients with high LAPTM4B-35 expression was 5.3 years (CI [0.32;0.89]) and the median OS of patients with low LAPTM4B-35 expression was 9.95 years (CI [1.12;3.13]). Examination of DFS, however, revealed a significantly worse outcome for patients with high LAPTM4B-35 expression (p = 0.0379 [Log Rank], p = 0.1421 [Gehan-Breslow]) (Fig. 2). The median DFS in the LAPTM4B-35 high group was 10.23 years whereas the LAPTM4B-35 low group remained undefined.

**Figure 3:**
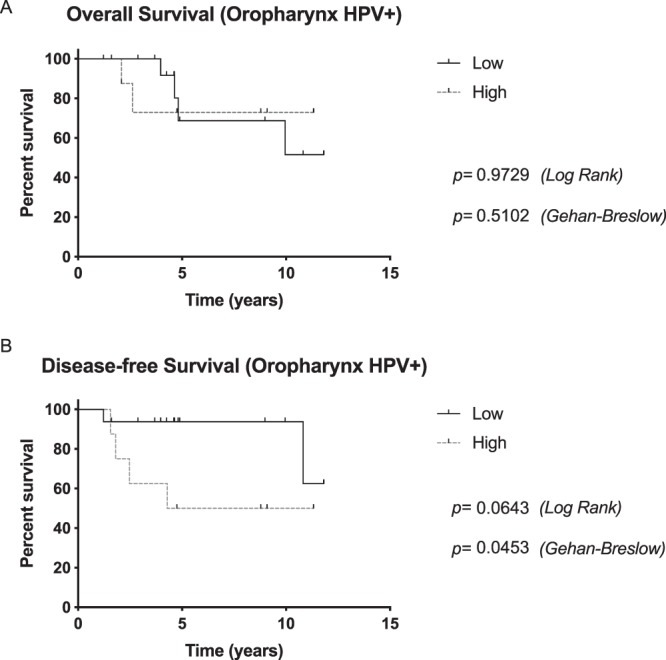
(**a**) Overall survival and (**b**) disease-free survival for patients with human papilloma virus (HPV) positive oropharyngeal carcinomas. High LAPTM4B-35 expression had a significantly negative influence on DFS in patients with HPV positive tumours.

Subsequently, OS and DFS were calculated for anatomical HNSCC subsites. For hypopharyngeal and laryngeal carcinoma the sample size was too small for statistical evaluation. In oral cavity carcinoma, expression of LAPTM4B-35 had no influence on OS (p = 0.4167 [Log Rank], p = 0.5947 [Gehan-Breslow]) or DFS (p = 0.7432 [Log Rank], p = 0.9303 [Gehan-Breslow]). In oropharyngeal carcinoma, high expression of LAPTM4B-35 almost correlated with a statistically significant worse DFS (p = 0.0545 [Log Rank], p = 0.0856 [Gehan-Breslow]). Since some oropharyngeal carcinomas are associated with HPV infection, we subsequently established Kaplan-Meier estimates stratified for HPV positive and HPV negative oropharyngeal carcinomas. The data showed that high LAPTM4B-35 expression had a significantly negative influence on DFS in patients with HPV positive tumours (p = 0.0643 [Log Rank], p = 0.0453 [Gehan-Breslow]) (Fig. 3). In HPV negative carcinomas, LAPTM4B-35 expression had no influence on OS (p = 0.8370 [Log Rank], p = 0.9806 [Gehan-Breslow]) or DFS (p = 0.6159 [Log Rank], p = 0.7349 [Gehan-Breslow]).

**Figure 4:**
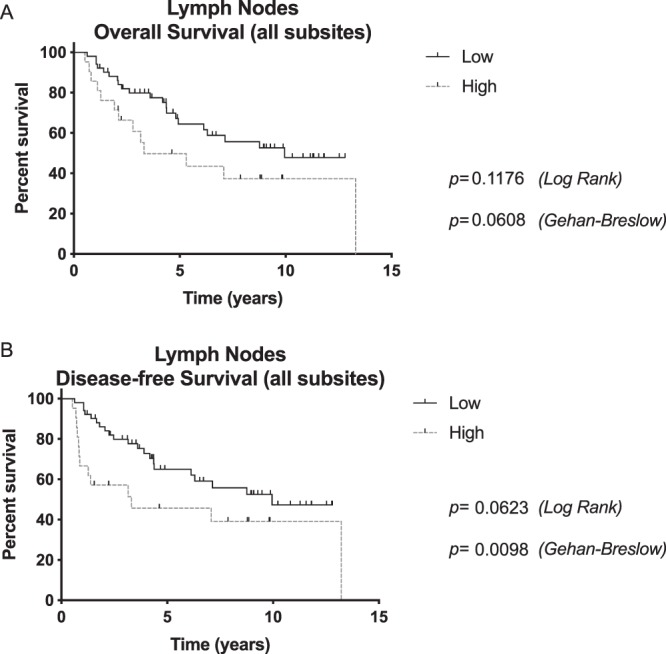
(**a**) Overall survival and (**b**) disease-free survival curves according to LAPTM4B-35 expression (high/low) in lymph node metastases. Patients with high LAPTM4B-35 expression in lymph nodes had a statistically significant worse DFS.

Finally, the impact of LAPTM4B-35 expression in lymph node metastases was evaluated. Patients with high LAPTM4B-35 lymph node expression had a statistically significant worse DFS (p = 0.0623 [Log Rank], p = 0.0098 [Gehan-Breslow]) (Fig. 4). OS was also worse in this patient group although levels of significance were not reached (p = 0.1176 [Log Rank], p = 0.0608 [Gehan-Breslow]).

Moreover, univariate and multivariate analyses were performed to assess if LAPTM4B-35 expression is an independent marker for recurrence and survival (Table 2). Hazard ratios in relation to LAPTM4B-35 expression, tumour localization, HPV infection, sex, age and classification (T and N) were calculated. Univariate analysis showed that high LAPTM4B-35 expression was associated with a significantly increased risk of recurrence (p = 0.041). In the multivariate analysis, however, LAPTM4B-35 expression association was not significant (p = 0.082).

**Table 2:** Factors predictive of mortality and recurrence in univariate and multivariate analyses.

	Univariate HR (95%CI)	*p*-value	Multivariate HR (95%CI)	*p*-value
**Mortality**				
LAPTM4B-35 high vs. low	1.34 (0.80–2.24)	0.256	N/A	N/A
Tumor localization	0.61 (0.36–1.05)	0.078	0.71 (0.37–1.33)	0.287
HPV positive vs. negative	0.54 (0.25–1.15)	0.112	0.75 (0.31–1.79)	0.522
Sex	1.14 (0.60–2.16)	0.671	N/A	N/A
Age	1.05 (0.63–1.78)	0.826	N/A	N/A
T classification	2.43 (1.41–4.16)	0.001	2.01 (1.12–3.58)	0.018
N classification	1.41 (0.83–2.39)	0.196	1.47 (0.85–2.54)	0.168
**Recurrence**				
LAPTM4B-35 high vs. low	1.92 (1.02–3.59)	0.041	1.7 (0.93–3.32)	0.082
Tumor localization	0.56 (0.29–1.08)	0.084	0.63 (0.32–1.24)	0.187
HPV positive vs. negative	0.82 (0.37–1.79)	0.619	N/A	N/A
Sex	1.52 (0.67–3.48)	0.313	N/A	N/A
Age	1.01 (0.54–1.90)	0.954	N/A	N/A
T classification	1.62 (0.81–3.24)	0.167	1.36 (0.67–2.77)	0.383
N classification	1.09 (0.58–2.05)	0.765	N/A	N/A

## Discussion

Treatment of head and neck squamous cell carcinoma is currently a therapeutic challenge. Therapeutic options available are often accompanied by significant side effects that lead to a reduction in quality of life^28^. The identification of new molecular biomarkers that predict patient response and outcome could help to distinguish the intensity of therapy applied in different patient subsets.

Expression of the LAPTM4B-35 oncogene has been shown to be a negative prognostic factor in a variety of malignant tumours^29^. This study is the first to investigate the HNSCC expression of LAPTM4B-35 in primary tumours and metastasis on the protein level. Within the primary tumours, immunohistochemical analysis showed that LAPTM4B-35 was expressed at low levels in 53.6% of the HNSCC tumours and highly expressed in 46.4%. In contrast, healthy adjacent tissue showed no staining. Variable LAPTM4B-35 expression patterns are common in other types of carcinoma. High expression of LAPTM4B-35 has been reported, for example, in 62.5% of colorectal carcinoma^17^, 56.3% of breast cancer^13^ and 70.91% of endometrial carcinoma^14^ patients.

In 29.2% of the corresponding lymph node metastases, LAPTM4B-35 was expressed at a high intensity. This is in contrast to a study on axillary lymph nodes of breast cancer patients where high LAPTM4B-35 expression was found in 19 of 20 (95%) lymph node metastases^30^.

Moreover, LAPTM4B-35 expression correlated with T and N classifications and UICC stage. High LAPTM4B-35 expression is also associated with tumour stage in a number of other carcinomas such as breast cancer, hepatocellular carcinoma and gastric carcinoma^31–33^. Correlation with clinicopathologic characteristics revealed that LAPTM4B-35 overexpression is associated with a significantly poorer DFS and a higher recurrence rate. These data are consistent with previously reported results for other types of cancer. In colorectal carcinoma, patients with high LAPTM4B-35 expression had a worse OS and DFS^17^.In non-small-cell lung cancer, LAPTM4B-35 overexpression was associated with significantly worse 5-year OS and progression free survival^34^. Moreover, high LAPTM4B-35 expression positively correlated with worse OS and progression free survival in breast cancer^13^. Similar results were found in glioblastoma, gastric cancer, prostate cancer, hepatocellular carcinoma and endometrial carcinoma^14,16,18,35–37^. However, these studies did not correlate LAPTM4B-35 expression in lymph node metastases with patient survival data. This study shows that high nodal expression of LAPTM4B-35 in HNSCC is associated with a statistically significant worse DFS.

Head and neck tumours occur in distinct anatomical regions. In the oropharynx, a subset of carcinomas is related to HPV infection associated with a better prognosis^38,39^. We therefore correlated expression of LAPTM4B-35 with survival data of each anatomical region and HPV status in oropharyngeal carcinoma. The analysis showed that patients with HPV positive oropharyngeal cancer and a high LAPTM4B-35 expression had a significantly worse DFS. In cervical carcinomas, which in most cases are associated with HPV infection, high expression of LAPTM4B-35 was also related to poor OS and DFS^16^. A study with the cervical cancer cell line HeLa demonstrated that RNAi-mediated knockdown of LAPTM4B-35 inhibited proliferation, invasion and angiogenesis *in vitro*. Western blot analysis revealed that LAPTM4B-35 downregulation decreased the levels of vascular endothelial growth factor, matrix metalloproteinase (MMP)-2, MMP-9, cyclin-dependent kinase 12 and hypoxia-inducible factor 1-α expression^40^.

In conclusion, this study examines for the first time the expression of LAPTM4B-35 in HNSCC tumour samples and in associated lymph node metastases at the protein level. Since high LAPTM4B-35 expression is associated with advanced tumour stage and significantly worse DFS as well as a higher recurrence rate, LAPTM4B-35 could serve as a negative prognostic marker in patients with HNSCC.

## Materials and Methods

### Patients

In total, 127 patients with freshly diagnosed HNSCC were included in the study. The study cohort was assembled by our research group as described previously^41^. All patients were treated with surgery and adjuvant radiotherapy at the Medical University of Vienna in the period between 2002 and 2012. Exclusion criteria were a second primary carcinoma, previous irradiation in the head and neck area and external treatment. Prior to treatment, all patients were presented to the institutional multidisciplinary tumour board. In some cases (i.e. extracapsular spread, perineural invasion), chemotherapy was administered additionally after surgery. This study was approved by the ethics committee of the Medical University of Vienna (EK 1311/2018). All subjects participating in this study gave written informed consent. All tests were performed in accordance with the Declaration of Helsinki and the guidelines for good scientific practice of the Medical University of Vienna.

### Tissue microarray construction and immunohistochemistry

Formalin-fixed paraffin-embedded HNSCC specimens obtained from surgically resected specimens were selected. TMA were then constructed using a Galileo TMA CK Series-HTS Tissue computer assisted Microarray Platform (Integrated Systems Engineering Srl, Milan, Italy) as described previously^42^. Samples measured 2 mm in diameter and 4–6 mm in length. Haematoxylin and eosin (H&E) staining was performed to verify histology. Immunohistochemical staining was performed using the Lab Vision Ultra V Block kit (Thermo Fisher Scientific, Waltham, MA, USA) and the Lab Vision Ultravision LP detection system (Thermo Fisher Scientific) according to the manufacturer’s protocol^41^. Colon sections were stained as positive controls^17^ to determine the appropriate antibody dilution and antigen retrieval buffer. Citrate buffer (pH 6.0) was used for antigen retrieval in the microwave and the anti-LAPTM4B-35 antibody (Abcam, Cambridge, United Kingdom) was diluted 1:200. Slides were incubated for one hour at room temperature. For negative controls, primary antibody was replaced by rabbit immunoglobulin G isotype control (Abcam). Samples were analysed using an Olympus BH-2 microscope (Olympus, Tokyo, Japan).

Based on the intensity of the cytoplasmic staining of neoplastic cells, all samples were assigned to one of four categories (negative, weak, moderate or strong). Analysis was performed by an experienced pathologist (F.O.). The percentage of stained neoplastic cells was then recorded and biopsies were divided into LAPTM4B-35 positive (>10% of cells staining positive) or LAPTM4B-35 negative (<10% of cells staining positive) groups. For statistical analysis, we combined patients with negative and weak expression of LAPTM4B-35 in the group of “LAPTM4B-35 low” patients and patients with moderate and strong expression in the group of “LAPTM4B-35 high” patients.

### Statistical analysis

Descriptive statistics were used to report clinical data. To compare categorical data between two groups, the Fisher exact test was used. The chi square test was applied when evaluating three or more groups. OS and DFS were calculated using the Kaplan-Meier method. The log-rank (Mantel-Cox) and Gehan-Breslow-Wilcoxon tests were used to compare statistical differences between the established patient groups. Hazard ratios with 95% confidence intervals (CI) were calculated to reflect a significance level of 0.05. Univariate logistic regression was used to assess the effects of demographic (age, sex) and tumour-specific (LAPTM4B-35 expression, HPV status, UICC stage, T & N classification, localization of the primary lesion) characteristics on OS and DFS. Variables with p < 0.2 in the univariate analyses were included in the multivariate logistic regression analysis. Multivariate analyses were performed using the Cox regression model to identify independent variables. P-values < 0.05 were determined as statistically significant. For data analysis and visualization, SPSS software (Version 21.0; SPSS Inc., Chicago, IL, USA) and GraphPad Prism software (GraphPad Software Inc., La Jolla, CA, USA) were used.
